# A Urea Potentiometric Biosensor Based on a Thiophene Copolymer

**DOI:** 10.3390/bios7010013

**Published:** 2017-03-03

**Authors:** Cheng-Yuan (Kevin) Lai, Peter J. S. Foot, John W. Brown, Peter Spearman

**Affiliations:** Materials Research Centre & School of LSPC, Faculty of Science, Engineering and Computing, Kingston University London, Penrhyn Road, Kingston upon Thames, Surrey KT1 2EE, UK; kevinla.lai@msa.hinet.net (C.-Y.L.); j.brown@kingston.ac.uk (J.W.B.); p.spearman@kingston.ac.uk (P.S.)

**Keywords:** urea, urease, biosensors, potentiometry, polythiophene, conducting polymer

## Abstract

A potentiometric enzyme biosensor is a convenient detector for quantification of urea concentrations in industrial processes, or for monitoring patients with diabetes, kidney damage or liver malfunction. In this work, poly(3-hexylthiophene-*co*-3-thiopheneacetic acid) (P(3HT-*co*-3TAA)) was chemically synthesized, characterized and spin-coated onto conductive indium tin oxide (ITO) glass electrodes. Urease (Urs) was covalently attached to the smooth surface of this copolymer via carbodiimide coupling. The electrochemical behavior and stability of the modified Urs/P(3HT-*co*-3TAA)/ITO glass electrode were investigated by cyclic voltammetry, and the bound enzyme activity was confirmed by spectrophotometry. Potentiometric response studies indicated that this electrode could determine the concentration of urea in aqueous solutions, with a quasi-Nernstian response up to about 5 mM. No attempt was made to optimize the response speed; full equilibration occurred after 10 min, but the half-time for response was typically <1 min.

## 1. Introduction

Enzyme-based electrochemical biosensors have achieved great commercial importance since the first use of glucose oxidase in an amperometric sensor for glucose [1] in 1962. All such systems require the incorporation of the biocatalytic element onto (or into) the sensing electrode structure, and this has been achieved by physical adsorption [2] or entanglement [3], DNA intercalation [4] and a wide variety of covalent bonding techniques [5,6,7,8].

Conducting polymers may be considered as good transducers which can help to convert biochemical signals into electronic signals in enzyme biosensors [9]. They have the important advantage of being mixed conductors (allowing both electronic and ionic/molecular transport) [10] as well as having greater biocompatibility than many inorganic transducers. Immobilization of an enzyme stably onto conducting polymer electrodes is an important type of technology for the fabrication of efficient and enduring enzyme biosensors [11,12]. Electrochemical co-deposition has been utilized widely to entrap enzymes into polypyrrole or its derivatives during the process of polymerization [9,12,13,14,15]. However, the effects of harsh chemical conditions on covalent linkages within the enzyme proteins can result in denaturation of the active material. Alternatively, immobilizing the enzyme directly onto the conducting polymers can avoid the enzyme experiencing aggressive conditions. The carbodiimide coupling reaction provided by 1-ethyl-3-(3-dimethylaminopropyl) carbodiimide hydrochloride (EDC) and N-hydroxysuccinimide (NHS) has previously been used to create a peptide bond between amides and carboxylic acids in aqueous solution at room temperature [16]. This method has been applied for the immobilization of enzymes on modified conducting polymers under moderate conditions [17].

The concentration of urea in blood serum is used to monitor diabetes and also to indicate the onset of kidney failure and liver malfunction. A potentiometric conducting polymer biosensor is a convenient device to quantify urea concentration, since its rest potential varies when urea is hydrolyzed by urease, causing a p[OH^−^] change in the analyte [9,12]. Many studies have investigated the immobilization of enzymes on polypyrrole because of its high biocompatibility and low electropolymerization potential [11,17,18,19,20,21]. However, a drawback is the frequent poor morphology of polypyrrole film, which may cause the detection to be irreproducible; the polymer is also susceptible to oxidative damage. Other conducting polymers such as polyaniline show more stable biosensor properties [22], and have been used in potentiometric [23] and amperometric [24] urea biosensors, but polyaniline has potential toxic hazards. A copolymer of glycidyl methacrylate and vinyl ferrocene [25] has shown some promise for urea electroanalysis.

Polythiophenes have been comparatively neglected in the literature on electrochemical conducting polymer biosensors, although a glucose sensor based on polythiophene was reported as long ago as 1996 [26]. This neglect may have been due to the high electropolymerization potential of thiophenes and the low conductivity of polythiophenes under the typical conditions of biosensor use, but some polythiophene-based sensors for glucose [27,28,29,30,31], lactate [32,33], choline [34], glutamate [35], ascorbic acid [36] and H_2_O_2_ [37,38] have successfully been produced.

**Scheme 1:**
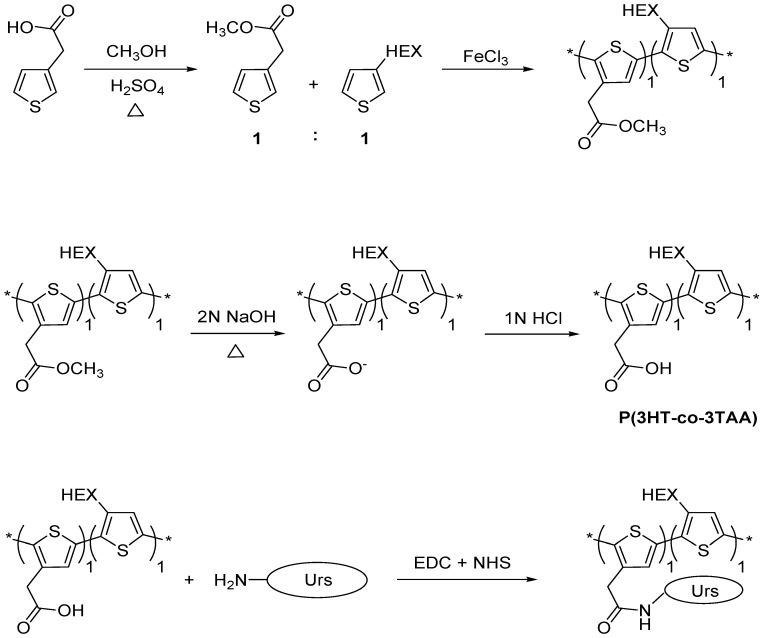
Synthesis of poly(3-hexylthiophene-*co*-3-thiopheneacetic acid 1:1) (P(3HT-*co*-3TAA)) and its functionalization with urease (Urs) by formation of a peptide linkage. EDC: 1-ethyl-3-(3-dimethylaminopropyl) carbodiimide hydrochloride; NHS: N-hydroxysuccinimide; Urs: urease.

In this paper, a conducting copolymer, poly(3-hexylthiophene-*co*-3-thiopheneacetic acid 1:1) (P(3HT-*co*-3TAA)) was synthesized. Urease was covalently attached to the carboxylate groups of P(3HT-*co*-3TAA) through the amine functionalities in the aminoacids, via carbodiimide coupling (Scheme 1), and the urease activity was investigated by spectrophotometric response studies.

The modified polymer was used to produce urease (Urs) electrode Urs/P(3HT-*co*-3TAA)/ITO glass) biosensors, which were found to give a quasi-Nernstian response to urea concentrations up to about 5 mM by potentiometric assay. This urease electrode could therefore be used to monitor the level of urea in blood serum, which is typically 1.3–3.5 mM (8–20 mg/dL) [8].

## 2. Materials and Methods

### 2.1. Chemicals

The starting materials were all from Sigma-Aldrich, St. Louis, MO, USA (supplied as Aldrich or Fluka products, as indicated below). The grades/purities were as follows: 3-hexylthiophene (Aldrich 99%), 3-thiopheneacetic acid (Aldrich, 98%), anhydrous methanol (Aldrich, 99%), anhydrous iron (III) chloride (Aldrich, 98%), urease (Fluka BioChemika, obtained from Jack beans; activity 100 units mg^−1^), *N*-(3-dimethylaminopropyl)-*N*’-ethyl-carbodiimide hydrochloride (ECD, Fluka 98%), urea (Aldrich, ≥99.5%), TRIS buffer (tris(hydroxymethyl)aminomethane hydrochloride) (Aldrich), thionyl chloride (Aldrich, +99%), 1,6-diaminohexane (Aldrich, 98%), potassium aluminum sulfate dodecahydrate (Fluka, ACS puriss.) and N-hydroxysuccinimide (NHS, Fluka 97%).

### 2.2. Synthetic Procedures

#### 2.2.1. Esterification of 3-thiopheneacetic acid

Firstly, 3-thiopheneacetic acid (1.42 g, 0.01 mol) was dissolved in methanol (20 mL), followed by the addition of a few drops of conc. H_2_SO_4_. The solution was stirred for 24 h at 100 °C, and the excess methanol was then removed by rotary evaporation. Distilled water (10 mL) was poured into the flask and the shaken mixture was extracted with diethyl ether (10 mL). The organic layers were collected and dried with anhydrous MgSO_4_, and the solvent was removed by rotary evaporation. The methyl ester product was a yellow oily liquid showing a single component with *m*/*z* = 156 by gas chromatography-mass spectrometry (GC-MS); yield 1.5 g (96%; 9.6 mmol).

#### 2.2.2. Synthesis of poly(3-hexylthiophene-*co*-methyl 2-(thiophene-3-yl)acetate), (P(3HT-*co*-MTA))

Firstly, 3-hexylthiophene (1.34 g, 8.0 mmol) and methyl 2-(thiophene-3-yl)acetate (MTA) (1.24 g; 8.0 mmol) were dissolved in CHCl_3_ (20 mL) with FeCl_3_ (5.19 g, 32 mmol) and stirred for 24 h at 0 °C under nitrogen atmosphere. The solution was poured into methanol (100 mL) and left for 1 h to form a precipitate. After filtration, the solid was collected on filter paper and the FeCl_3_ was removed by washing with methanol in a Soxhlet extractor for 8 h. The product was dried at 60 °C under reduced pressure for 24 h. The yield of dark-brown powder, poly(3-hexylthiophene-*co*-methyl 2-(thiophene-3-yl)acetate (P(3HT-*co*-MTA)), was 1.44 g (56%). Proton nuclear magnetic resonance (^1^H-NMR) (400 MHz, CDCl_3_): δ = 6.96, 3.80, 3.73, 3.69, 2.78, 2.53, 2.20, 1.67, 1.41, 1.32, and 0.88 ppm. Fourier-transform infrared (FT-IR): 2953, 2923, 2855, 1743, 1516, 1457, 1434, 1377, 1329, 1259, 1197, 1168, 1017, 890, 831 and 725 cm^−1^.

#### 2.2.3. Hydrolysis of poly(3-hexylthiophene-*co*-methyl-2-(thiophene-3-yl) acetate)

P(3HT-*co*-MTA) (0.5 g) was added to a fivefold excess of 2 M NaOH solution and refluxed at 100 °C for 24 h. The resulting solid was suspended homogeneously in the solution under vigorous stirring, and 1 M HCl was added until the pH became less than 2. The solid was filtered off and dried at 80 °C under reduced pressure for 24 h. The brown powder product, poly(3-hexylthiophene-*co*-3-thiopheneacetic acid) (P3HT-*co*-3TAA) (0.36 g, 0.72%) was characterized by ^1^H-NMR and FT-IR spectroscopy. ^1^H-NMR (400 MHz CDCl_3_): δ = 6.93, 2.71, 2.50, 1.63, 1.55, 1.36, 1.26, 1.20, 0.82, and 0.00 ppm. FT-IR: 3437, 2954, 2923, 2855, 1713, 1516, 1463, 1377, 1260, 1223, 1099, 1051, 829, 724 cm^−1^.

#### 2.2.4. Immobilization of Urease on the Surface of P(3HT-*co*-3TAA) Using a Carbodiimide Coupling Reaction

P(3HT-*co*-3TAA) (0.10 g) dissolved in chloroform (5 mL) was spin-coated onto ITO glass (20 mm × 15 mm) and dried in an oven at 60 °C under reduced pressure for 24 h; the thickness of the film was typically about 400 ± 100 nm, as estimated from the intensity of the ultraviolet-visible (UV-vis) absorption peak. The ITO glass was dipped into a TRIS-HCl buffer solution (50 mM, pH = 7) containing urease (1 mg/mL). ECD (0.0573 g, 0.3 mmol) and NHS (0.0693 g, 0.6 mmol) were added to the buffer solution slowly and stirred for 3 h. Thus-modified ITO electrodes were washed with TRIS-HCl buffer (2 mL), dried at room temperature and stored in a freezer (−18 °C).

### 2.3. Spectrophotometric Assay of the Urease Immobilized on the Surface of P(3HT-co-3TAA)

**Scheme 2:**
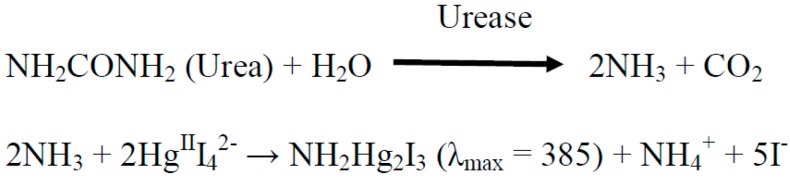
The reactions forming ammonia and its complex with Nessler’s reagent.

Using Nessler’s reagent to form a complex with ammonia products is a well-known method to assay the activity of urease. By the enzymatic hydrolysis of urea (Scheme 2), ammonia is ultimately produced, and is reacted with Nessler’s reagent (K_2_Hg^II^I_4_). The absorption of the amide complex NH_2_Hg_2_I_3_ at 385 nm is observed by UV-visible spectroscopy.

A test for possible leaching of urease from the sensor film (Urs/copolymer/ITO glass) was set up by dipping an electrode into TRIS-HCl buffer solution (1.0 mM; pH = 7) (5 mL) and shaking for 20 min. The electrode was removed from the solution and then urea solution (10 mM; 1 mL) with Nessler’s reagent (200 μL) was added. If there were any urease leaching from the film, an absorption peak of the amide complex would be found at 385 nm in the UV-vis spectrum; no such absorption was detected.

The response time of a sensor was investigated by determining the profile of its UV-visible absorbance against time. Firstly, six sample vials, each containing 5 mM urea solution, were marked with specific times (1.5, 2, 2.5, 3, 4, 5 and 6 min). The Urs/copolymer/ITO slice was dipped into each solution for the corresponding time and then removed. Nessler’s reagent (200 μL) was then added to each vial, and the solutions were left for 30 min. The sample absorbances were then measured at 385 nm vs. an ITO glass blank.

The lifetime of urease immobilized on the copolymer and stored at −18 °C was examined using the same method. The Urs/copolymer/ITO electrode was placed into 5 mM urea solution for 10 min after storage in the freezer for each month. Nessler’s reagent (200 μL) was added after the Urs/copolymer/ITO electrode was removed, and the solution absorbance at 385 nm was measured.

### 2.4. Potentiometric Assay Using the Electrochemical Biosensor System

For the potentiometric experiments, the Urs/copolymer/ITO glass was the working electrode, with a Pt counter electrode and an Ag/AgCl reference electrode. Three sensor electrodes were immersed in 1.0 M KCl (40 mL) and stirred slowly; the rest potential was measured until it had become stable for 5 min. Then, 1.0 M urea solution (40 μL) was added to the electrolyte (giving a urea concentration in the solution of 0.99 mM). The rest potential was measured each minute for 10 min. Urea was also prepared in the following concentrations: 2.99, 3.98, 4.97, 5.96 and 6.95 mM, and the rest potentials were measured for each solution.

### 2.5. Electrochemical Analysis by Cyclic Voltammetry

Urs/copolymer/ITO glass and copolymer/ITO glass (20 mm × 15 mm) working electrodes were prepared. A platinum counter electrode and a sealed aqueous Ag/AgCl/3.4 M KCl reference electrode (eDAQ type ET072-1) were used. The cyclic voltammetry experiments were run in a 0.1 M LiClO_4_/propylene carbonate electrolyte. The scan region was from −1000 mV to 2000 mV, starting from the rest potential, and a non-aqueous solvent was required in order to permit such a wide potential range; acetonitrile was found to give very erratic results, so propylene carbonate was chosen as the solvent. The cycles began in the positive direction, and the scan rate was 10 mV·s^−1^.

### 2.6. Polymer Film Morphology

Spun films of the copolymers P(3HT-*co*-MTA), P(3HT-*co*-TAA) and Urs-P(3HT-*co*-TAA) were examined by scanning electron microscopy using a Zeiss EVO 50 instrument with 20 kV accelerating potential after sputter-coating with Au-Pd. The films were remarkably smooth, and showed no discernible morphological features.

## 3. Results and Discussion

### 3.1. ^1^H-NMR Spectra for P(3HT-co-MTA) and P(3HT-co-3TAA)

**Figure 1:**
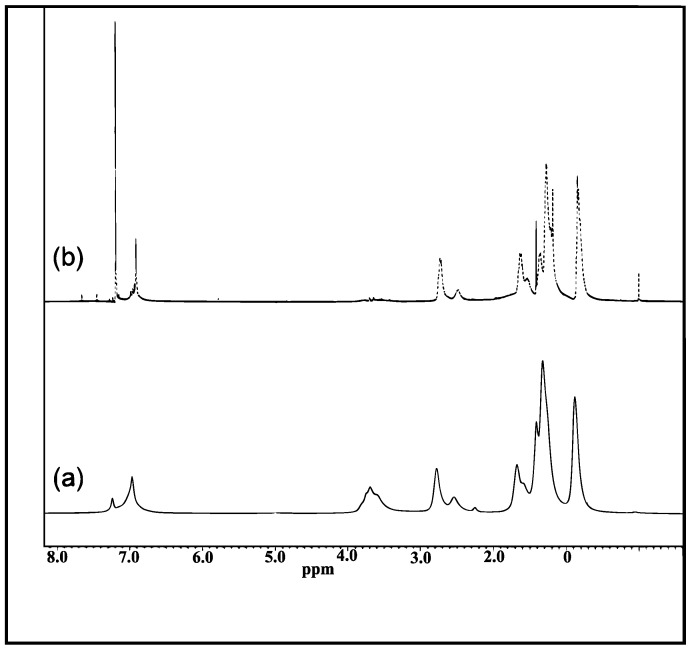
^1^H-NMR spectra of (**a**) poly(3-hexylthiophene-*co*-methyl 2-(thiophene-3-yl)acetate (P(3HT-*co*-MTA)) and (**b**) P(3HT-*co*-3TAA).

The ^1^H-NMR spectra confirmed the successful formation and hydrolysis of P(3HT-*co*-MTA). In the spectrum of that polymer (Figure 1a), the proton on the thiophene rings was observed at 6.97 ppm, and the peaks for –CH_2_ closest to the thiophene ring at 2.73 and 2.50 ppm. The -OCH_3_ resonance was at 3.70 ppm, and the other peaks at 1.63 ppm to 0.82 ppm were attributed to the protons on the alkyl groups. After acidification, the proton resonance on the thiophene ring became sharp and the -OCH_3_ peak vanished due to its being changed to -OH (Figure 1b). A new peak due to -COOH was observed at 0.00 ppm. Hence it was concluded that the synthesis of P(3HT-*co*-3TAA) had been achieved by copolymerizing 3-hexylthiophene and MTA and then hydrolyzing the product.

### 3.2. FT-IR Spectra of P(3HT-co-MTA), P(3HT-co-3TAA) and Urs/P(3HT-co-3TAA)

**Figure 2:**
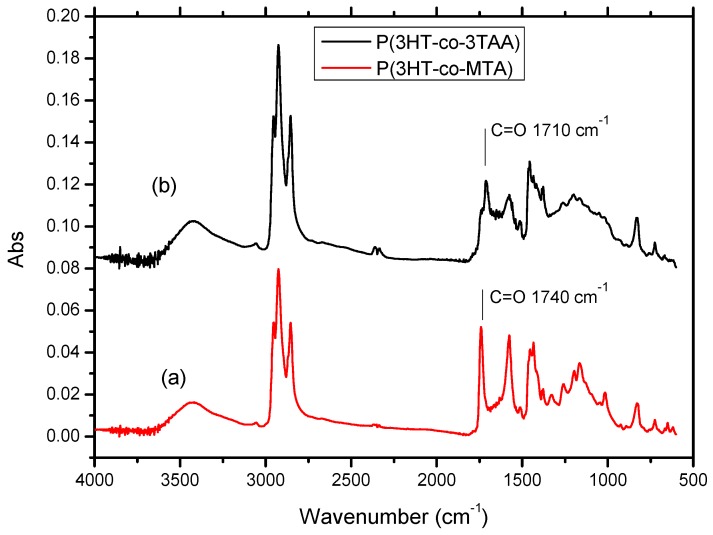
FT-IR spectra of copolymers (**a**) P(3HT-*co*-MTA); (**b**) P(3HT-*co*-3TAA).

In the FT-IR spectrum of the copolymer P(3HT-*co*-MTA) (Figure 2a), the C-H stretching vibrations absorbed at 2953, 2928 and 2854 cm^−1^. A strong peak due to C=O stretching was seen at 1740 cm^−1^, and vibrational modes of the thiophene ring at 1575, 1454 and 1377 cm^−1^. The C-O absorption of the acetate ester was at 1327 cm^−1^. It would be expected that the –OH stretching vibration at 3400 cm^−1^ would be undetectable in the spectrum of P(3HT-*co*-MTA), but some inadvertent moisture was present in the KBr disk samples, and therefore an –OH stretching peak was observed. After the acidification of P(3HT-*co*-MTA), the C=O stretching vibration shifted to 1710 cm^−1^ and the C-O stretch of the ester vanished from the spectrum of P(3HT-*co*-3TAA) (Figure 2b). It was thus concluded that the acetate ester in the copolymer had been converted to -COOH.

**Figure 3:**
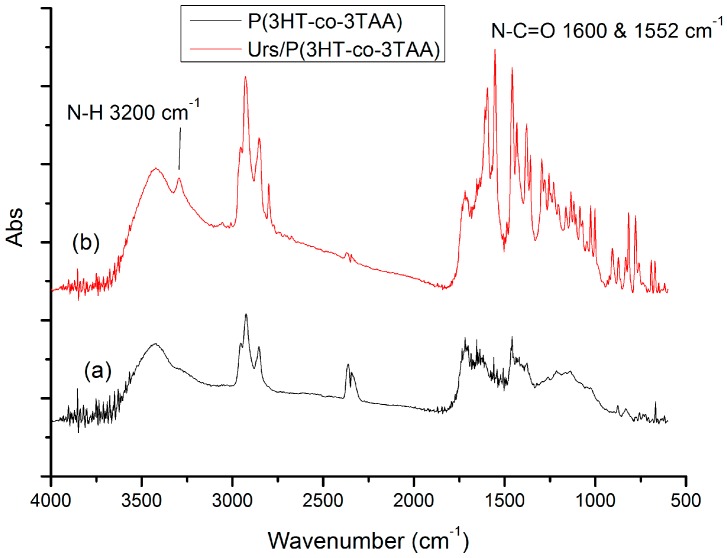
FT-IR spectra of (**a**) P(3HT-*co*-3TAA); (**b**) Urs/P(3HT-*co*-3TAA).

In the FT-IR spectrum of urease immobilized on the P(3HT-*co*-3TAA) by peptide bonds (Figure 3b), there are very strong peaks for N-C=O stretching vibrations, the symmetric and asymmetric deformations being seen at 1600 and 1552 cm^−1^. Furthermore, a very sharp peak appeared at 3200 cm^−1^, characterizing the N-H stretching vibration. Therefore, it can be concluded that a peptide bond between urease and (3HT-*co*-3TAA) was created via the carbodiimide coupling reaction.

### 3.3. Cyclic Voltammetry (CV) of P(3HT-co-3TAA) and Urs/P(3HT-co-3TAA) in 0.1 M LiClO_4_/Propylene Carbonate

**Figure 4:**
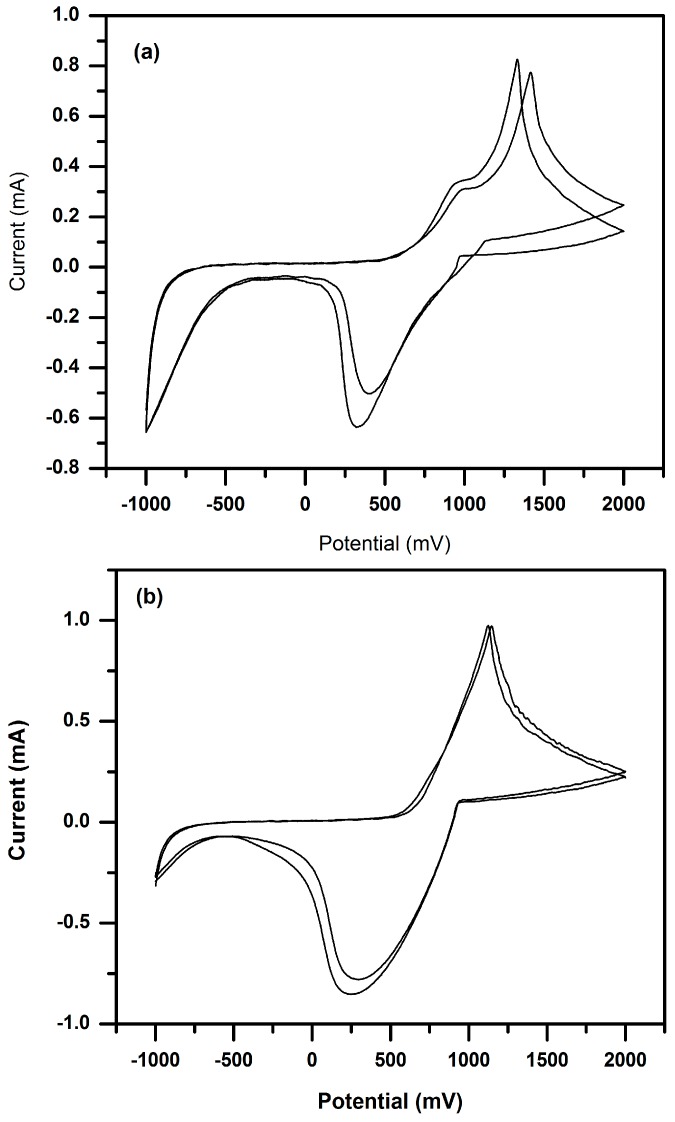
Cyclic voltammetry (CV) plots of (**a**) P(3HT-*co*-3TAA); (**b**) Urs/P(3HT-*co*-3TAA) in 0.1 M LiClO_4_/PC (Potentials are vs. Ag/AgCl/3.4 M KCl reference.).

In the cyclic voltammogram of P(3HT-*co*-3TAA) (Figure 4a), the main oxidation and reduction peaks appeared at +1390 and +300 mV respectively in the first cycle, and at +1290 and +390 mV respectively in the second and subsequent cycles. This electrochemical process appeared to be a partially-reversible doping-dedoping reaction. However, the observed reversibility increased after immobilizing urease on the surface of P(3HT-*co*-3TAA), and the peak potentials decreased somewhat, becoming about +1260 and +295 mV for oxidation and reduction respectively (Figure 4b). A possible reason is that the thiophene rings were held in a more planar configuration as a result of the urease functionalization. In addition, the enzyme-modified polymer lost the oxidation shoulders which are a notable feature in the CV of the unmodified copolymer.

It will be seen in Section 3.6 that the potential range associated with the biosensing action of the enzyme-modified electrodes (+100~300 mV) is below the region associated with the above redox processes; this implies that the modified copolymer electrodes would be in a largely-undoped semiconducting state when functioning as a potentiometric biosensor. 

### 3.4. UV-Visible Absorption Spectra of P(3HT-co-3TAA) and Urs/P(3HT-co-3TAA)

**Figure 5:**
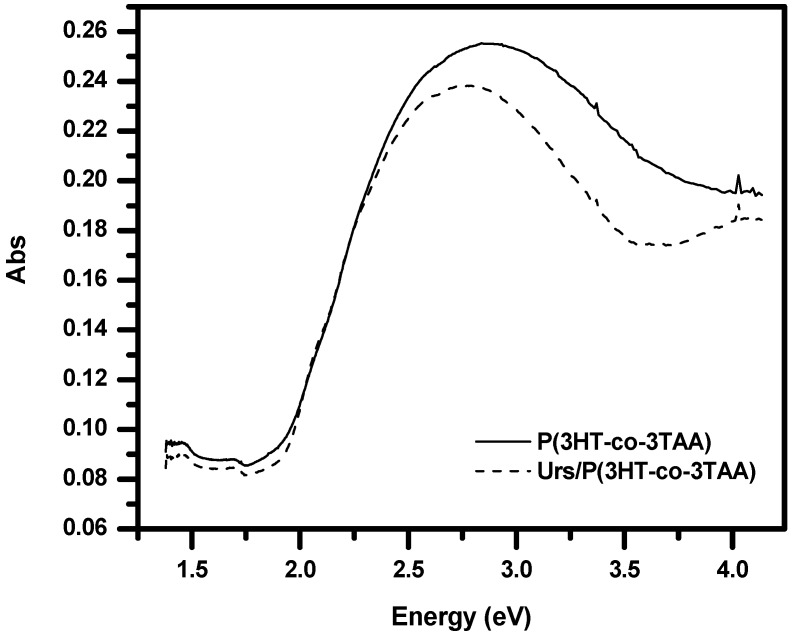
UV-visible absorption spectra of P(3HT-*co*-3TAA) and Urs/P(3HT-*co*-3TAA).

The π-π^*^ transition peak of a spin-coated film of P(3HT-*co*-3TAA) on ITO glass was observed at about 437 nm (2.84 eV) (Figure 5). After immobilizing urease on the film, the π-π^*^ transition shifted slightly to 453 nm (2.74 eV). Such a shift suggests that the P(3HT-*co*-3TAA) was slightly better conjugated as a result of coupling with the urease, presumably due to the thiophene units being forced into a more coplanar configuration as a result of successful attachment of urease to the P(3HT-*co*-3TAA).

### 3.5. Response Time of Urs/P(3HT-co-3TAA)/ITO Glass by Spectrophotometric Studies

**Figure 6:**
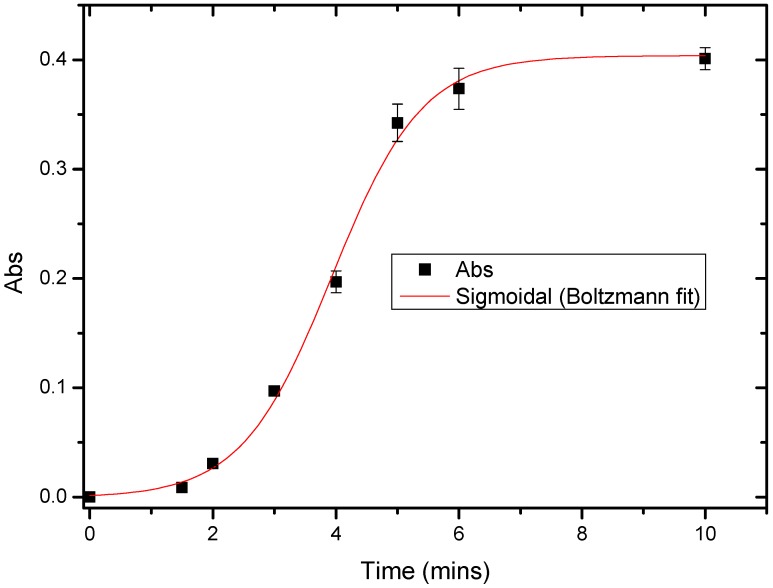
Absorbance (385 nm) vs. time for Urs/P(3HT-*co*-3TAA)/ITO glass assayed by Nessler’s reagent in 5 mM urea solution.

To observe the kinetic behavior of urease immobilized on the P(3HT-co-3TAA), the variation of absorbance was measured as a function of time during the enzyme-catalyzed hydrolysis of 4.97 mM urea solution. In Figure 6, the first absorbance was obtained at 1.5 min, and then increased monotonically with time until a plateau was reached.

The delay from 0 to 1.5 min suggests that the urea was initially overcoming the hydrophobic barrier of P(3HT-*co*-3TAA). After this point, the urease was functional until the urea had been hydrolyzed completely after about 6 min. Therefore it was concluded that in this biosensor system, the reaction required about six minutes to reach a steady state.

### 3.6. The Potentiometric Assay of Urease/P(3HT-co-3TAA)/ITO Glass

**Figure 7:**
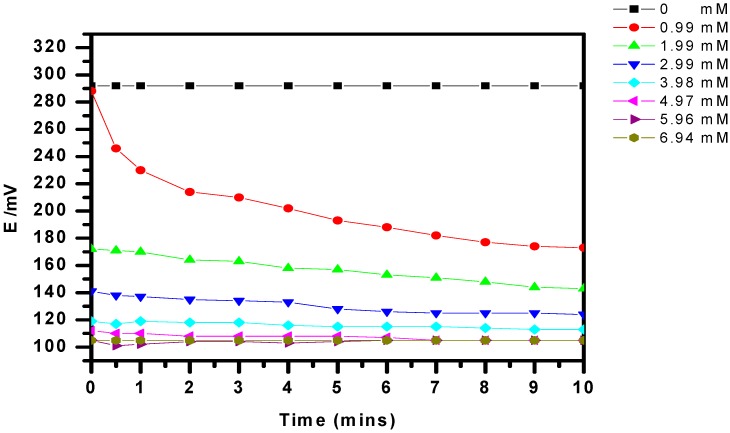
Evolution of electrode potential (vs. Ag/AgCl) as a function of time for various concentrations of urea in non-buffered solutions.

To prove that urease/P(3HT-*co*-3TAA) 1:1 on ITO glass was functional as a biosensor, the potential variation was recorded while the urease reacted with different concentrations of urea. The resulting local change in the p[OH-] provided adequate verification that ammonia was produced, and therefore that the urease had been successfully immobilized on the P(3HT-*co*-3TAA). The average initial potential in a blank aqueous solution (40 mL) was 292 mV, and this decreased suddenly to 288 mV on adding 1 M urea solution (40 μL) (Figure 7). The potential reached an equilibrium value of 173 mV after 10 min, and diminished further if the concentration of urea was increased. However, this trend ceased when the concentration of urea reached about 5 mM.

According to Nernst’s formalism, the half-cell equation for a reduction potential can be expressed as:

**E_red_ = E°_red_ + (RT/nF)·ln[a_red_/a_ox_]**

where E_red_ is the half-cell reduction potential, E° is the standard half-cell potential, R is the universal gas constant (8.314 J·K^−^1·mol^−1^) and F is the Faraday constant (96,485 C·mol^−1^). T is the absolute temperature and n is the number of electrons transferred in the half-reaction. Since the change of the reduction potential is related to the variation of p[OH^−^] in the solution, the Nernst equation can be modified thus:

**E = E° + (RT/βnF)·ln(a_OH_^−^)**

where E is the potential difference between the working electrode and the reference electrode (Ag/AgCl), **β** is the electron transfer symmetry factor and a_OH_^−^ is the activity of the hydroxide ion (equal to the concentration of OH^−^ multiplied by the activity coefficient α). Therefore, the potential can be expressed as:

**E = E° + (RT/βnF)·ln{[OH^−^]α}**

In addition, the variation of [OH^−^] results from the ammonia produced by the enzyme-hydrolysis of urea (Scheme 2). In view of the stoichiometry of that reaction, the Nernst equation can be modified to include the initial concentration of urea, thus:

**E = E° + (RT/βnF)·ln{2[urea]α}**

If α is assumed to be for ideal behavior (α = 1), and the equilibrated potential responses from 0.99 mM to 4.95 mM at 10 min (Figure 7) are used, the equation can be reduced to:

**y = −0.0426x − 0.0927 (R^2^ = 0.9987)**

**Figure 8:**
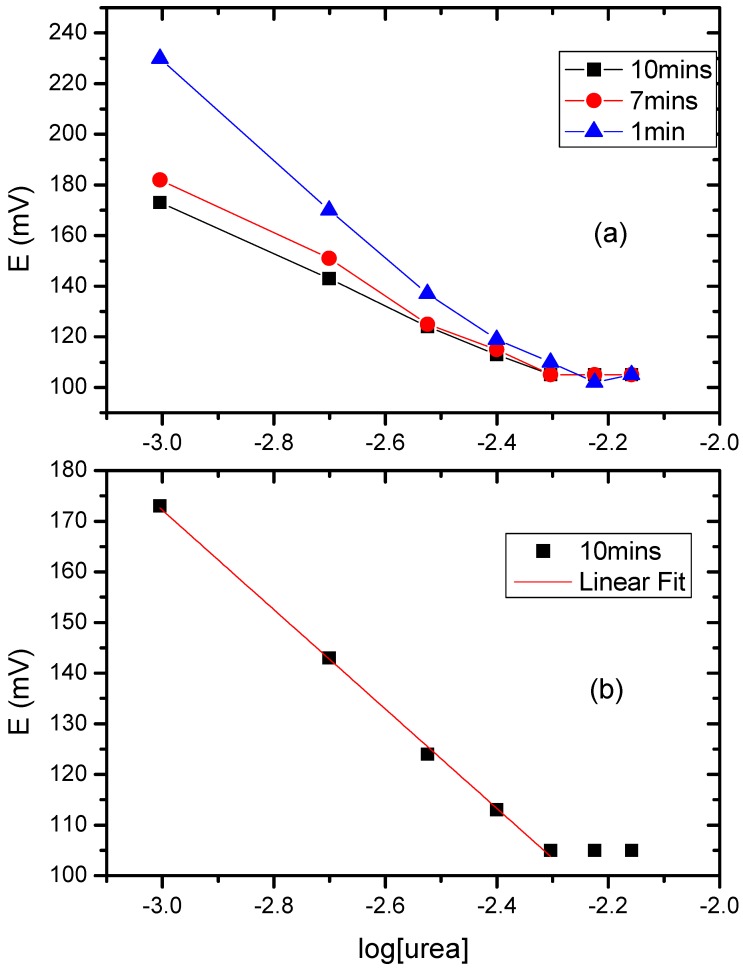
(**a**) Graph of electrode potential (w.r.t. Ag/AgCl) against log[urea] for several different delays from the moment of addition of urea; (**b**) Equilibrated data for the 10-min delay, showing linear fit in the region 1–5 mM.

For convenience, the average electrode responses are shown in Figure 8 as graphs of electrode potential (mV) vs. the decimal logarithm of [urea]/1 mM, so that the slope of the equilibrated graph in Figure 8b is −42.6 × 2.303 = −98.1 mV.

This implies that the value of RT/βnF was equal to 0.0426. A single electron transfer equilibrium was assumed because the variation of potential resulted from the change of p[OH^−^] in the analyte. Hence, the value of β in this half-reaction was calculated to be 0.598. The expected value of ln{2[urea]α} can be calculated from the ratio of ΔE and RT/βnF. The value of the activity coefficient (α) can be estimated from the ratio of ln{2[urea]α} values (Table 1) between the predicted and the measured values, although it should be noted that the numbers in parentheses are from data outside the linear region, and hence not really meaningful.

The experimental values were found to be quite close to the ideal ones in the region of urea concentration from 0.99 to 4.97 mM. As a result, it can be concluded that Urs/P(3HT-*co*-3TAA) successfully transduced a reliable potential variation whilst the urease enzyme hydrolyzed the urea. However, when the concentration of urea became greater than about 5 mM, this system could no longer be used as a urea biosensor because the potential response departed from a linear relationship with ln[urea].

## 4. Conclusions

The semiconducting thiophene copolymer P(3HT-*co*-3TAA) (1:1) was synthesized and used successfully in a urea biosensor, since it acts as a matrix that can immobilize urease on its surface. The covalent immobilization was carried out via the formation of peptide bonds, as confirmed by FT-IR spectroscopy. The redox peaks in the cyclic voltammograms and the shift of the π-π* optical transition offered indirect evidence of the successful bonding between P(3HT-*co*-3TAA) and urease. The Urs/P(3HT-*co*-3TAA)/ITO glass electrode reached equilibrium in a 4.97-mM urea solution after 6 min, as observed by spectrophotometry. The low conductivity of the thiophene polymer in its largely-undoped form would preclude the use of the electrode for reliable amperometric bioanalysis, but potentiometric response studies confirmed that this electrode could repeatedly detect the concentration of urea in aqueous solutions up to a maximum of about 5 mM. The normal level of urea in blood serum is in the region of 1.3 to 3.5 mM, and so the Urs/P(3HT-*co*-3TAA)/ITO glass electrode would be suitable for application in urea biosensors for blood serum analysis, or for process monitoring in situations where the analyte has a pH fairly close to neutral. The high intrinsic stability of polythiophenes, in comparison with many other common conducting polymers, would be a significant advantage in such applications.

## Figures and Tables

**Table 1:** Predicted and experimental values of ln{[urea]*α*2} and the activity coefficient (α). (T = 25.1 °C).

[urea] (mM)	E (V)	RT/βnF (*n* = 1)	ln{[urea]*α*2} (Predicted)	ln{[urea]*2}	α
0.99	0.182	0.0426	−6.3915	−6.2247	0.8462
1.99	0.152	0.0426	−5.6703	−5.5256	0.8660
2.99	0.125	0.0426	−5.0653	−5.1193	1.0555
3.98	0.115	0.0426	−4.8326	−4.8333	1.0007
4.97	0.105	0.0426	−4.5999	−4.6112	1.0113
5.96	0.105	0.0426	−4.5999	−4.4295	(0.8433)
6.95	0.105	0.0426	−4.5999	−4.2758	(0.6338)
